# Gene Expression and DNA Methylation as Prognostic Markers in Metastatic Castration-Resistant Prostate Cancer: Analysis of Circulating Tumor Cells and Paired Plasma-Derived Exosomes

**DOI:** 10.3390/cancers15225325

**Published:** 2023-11-08

**Authors:** Mohamed Ali Hussein, Gnanasekar Munirathinam

**Affiliations:** 1 Department of Pharmaceutical Services, Children’s Cancer Hospital Egypt 57357, Cairo 11562, Egypt; mohamedalihussien8554@gmail.com; 2 Department of Biology, School of Sciences and Engineering, American University in Cairo, New Cairo 11835, Egypt; 3 Department of Biomedical Sciences, College of Medicine, University of Illinois, Rockford, IL 61107, USA

## 1. Introduction

Prostate cancer (PCa) is the most prevalent cancer among men and is the second leading cause of cancer-related death in the United States [1]. PCa is initially innocuous in 80% of patients; the 5-year survival rate is 100%. The remaining 20% of patients will develop resistance to hormonal therapy and progress to a fatal status called castration resistance prostate cancer (CRPC), which eventually progresses to metastatic CRPC (mCRPC), for which the rate of 5-year survival rate dramatically declines to 26–30% [2]. Currently, the standard armamentarium for treating mCRPC includes the usage of chemotherapy (taxanes-based therapy such as docetaxel and cabazitaxel), antiandrogen therapy (abiraterone and enzalutamide), a radiopharmaceutical (radium-223), and the immunotherapy-based treatment (sipuleucel-T) [2,3,4]. The early detection of cancer combined with proper treatment options will substantially increase the rate of cured cancer patients. In fact, monitoring cancer progression is crucial and has garnered significant attention from the scientific community in the last decade. Recent advancements in liquid biopsy (LB) have paved the way for real-time monitoring, which not only aids in the early detection of cancer, but also helps to monitor tumor progression, recurrence, the development of resistance, and treatment responses. LB involves analyzing blood samples for reliable tumor-derived markers, such as circulating tumor cells (CTCs), circulating tumor DNA (ctDNA), methylation markers, extracellular vehicles (EVs), and tumor-educated platelets (TEPs). These markers hold tremendous potential for personalized medicine, as they provide valuable information for diagnosis as well as offer a new approach to aiding the treatment based on the change in the tumor molecular dynamics [5,6,7].

Unlike several protein biomarkers that have been used for aiding the diagnosis of cancer, such as a prostate-specific antigen, alpha-fetoprotein, carbohydrate antigen 19-9, and carcinoembryonic antigen, which lack specificity and possess a high rate of false positivity, tumor-derived biomarkers represent a promising strategy that could be used to explore the treatment efficacy and resistance development monitoring in real-time, such as CTCs and tumor-derived extracellular vesicles (tdEVs) [8]. CTCs are tumor cells relinquished from the primary tumor that extravasate into the blood. They are very rare and represent a set of primary tumor cells that possess phenotypic characteristics resulting from the epithelial-to-mesenchymal transition (EMT), which enables metastatic potential and form the seeds for distant metastatic colonies. CTCs can express epithelial markers, such as the epithelial cell adhesion molecule (EpCAM) and cytokeratin (CK); mesenchymal markers, such as N-cadherin; and tumor-specific markers, such as human epidermal growth factor receptor-2 (HER-2), estrogen receptor (ER), and prostate-specific membrane antigen (PSMA) [9,10,11,12,13]. Several technologies have been used to enrich the CTCs pool in blood based on physical or biological properties, such as CellSearch (CS), CTC-chip, and flow cytometry sorting [14].

Moreover, tdEVs can be classified into three main categories, including exosomes, microvesicles, and apoptotic bodies, depending on their size and molecular mechanism of release, which is particularly important for tumor development and progression [15,16,17]. Exosomes are a subclass of tdEVs, with the smallest ones being 30–100 nm, which are compared to the microvesicles and apoptotic bodies with sizes of 100–1000 nm and 800–5000 nm, respectively [15]. In regard to the release mechanism, exosomes mainly result from the fusion of late endosomes or multivesicular bodies and the plasma membrane, which contrasts the other subclasses that are released from the plasma membrane [15,18]. Several methods have been used to separate exosomes, including size exclusion chromatography, immunoaffinity, and microfluidics [19]. Furthermore, molecular characterization is critical to measure the gene expression patterns and epigenetic alteration of CTCs and plasma-derived exosomes for diagnostic, therapeutic, and prognostic purposes.

Recently, Zavridou et al. [20] conducted a comparative study emphasizing the alteration in the gene expression and DNA methylation pattern between the CTCs and exosomes and their significance as a prognostic marker in mCRPC. Their experimental study involved by collecting the peripheral blood of 62 mCRPC and 10 healthy donors (HD), followed by the isolation of the EpCAM-positive CTCs and exosomes. After that, RNA was extracted from the CTCs and exosomes, followed by cDNA synthesis and the examination of the gene expression pattern via RT-qPCR for several markers, including aldehyde dehydrogenase 1 (ALDH1); cytokeratins such as CK-8, CK-19, and CK-18; PSMA; TWIST family transcription factor 1 (TWIST1); programmed death-ligand 1 (PD-L1); androgen receptor-full length (AR-FL); AR splice variants: AR-V7 and AR-567. In addition, gDNA was extracted from both the CTCs and exosomes, followed by a treatment with sodium bisulfite (SB); after that, the authors examined the methylation pattern for several markers, such as Ras association domain family member 1 (RASSF1A), Glutathione S-transferase Pi (GSTP1), and SCHLAFEN 11 (SLFN11), using real-time methylation-specific PCR (Real-Time MSP). Moreover, CTC and tdEV enumeration were also performed.

The authors compared the aforementioned markers of the EpCAM-positive CTCs and exosomes in the healthy controls and mCRPC patients. Multiple comparisons helped to identify the features that were differentially expressed between the groups. The gene expression data revealed that the CK-8, CK-19, and CK-18 expression levels in the EpCAM-positive CTCs is more highly expressed in the mCRPC patients compared to those of the HDs. In the exosomes, the CK-8 and CK-18 expression levels were significantly higher in the mCRPC patients compared to those of the CK-19 patients, which are expressed in both the HD and mCRPC cohorts. Therefore, CK-19 was cons idered a non-specific marker in exosomes. The CK-8 and CK-18 expression levels were lower in the exosomes compared to those of the EpCAM-positive CTCs. In addition, the TWIST1, ALDH1, PSMA, and PD-L1 expression levels were significantly higher in the mCRPC patients compared to those of the HDs; the TWIST1, PSMA, and PD-L1 expression levels in the EpCAM-positive CTCs were higher than those of the exosomes in contrast to ALDH1. Moreover, AR-FL was found to be expressed at a higher level in the HDs as well as the mCRPC patients in both the EpCAM-positive CTCs and exosomes in comparison to its splice variants AR-V7 and AR-567, which were expressed only in the mCRPC patients with higher rates in the EpCAM-positive CTCs than those of the exosomes. However, using the chi-square (χ^2^) test, the authors revealed that the direct comparison of all the implicated markers, except for CK-19, between the EpCAM-positive CTCs and exosome markers were statistically insignificant among 62 mCRPC patients. Furthermore, the authors performed Kaplan–Meier analysis to examine the correlation between gene expression and overall survival (OS) for the CTCs and exosomes. The results show that in the EpCAM-positive CTCs, the expression levels of CK-19, TWIST1, and PSMA were significantly correlated with a lower OS rate compared to that of CK-8, which was the only marker significantly correlated with a low OS rate among the exosomes. Likewise, univariate analysis demonstrated that CK-19, TWIST1, and PSMA expression in the CTCs were significantly associated with a higher risk of death in the positive patients compared to the CK-8 patients, which was the only exosome marker associated with a higher death risk among the positive patients.

Furthermore, the authors investigated the variation in DNA methylation in the CTCs and exosomes for the HD and mCRPC samples, respectively. The authors revealed that the GSTP1, RASSF1A, and SLFN11 methylation results were positive in the mCRPC samples in both the EpCAM-positive CTCs and exosomes. Using the χ^2^ test, the direct comparison of the mCRPC samples revealed a positive correlation between GSTP1 and RASSF1A, and there is a concurrence of GSTP1, RASSF1A, and SLFN11 in both the EpCAM-positive CTCs and exosomes. GSTP1, RASSF1A and SLFN11 are more highly expressed in the exosomes as compared to the EpCAM-positive CTCs. In addition, GSTP1 and RASSF1A methylation in the plasma-derived exosomes were significantly associated with a lower OS rate and a high risk of death. In contrast, GSTP1 methylation was the only marker associated with a lower OS rate and an increased risk of death in the EpCAM-positive CTCs.

The authors also performed the Multivariate Cox Regression Analysis for CK-19, TWIST1, PSMA, and GSTP1 methylation in the CTCs for the mCRPC cohort. They revealed that those markers have a significant prognostic potential for diagnosing patients with mCRPC, apart from the Gleason score or CTC count.

CTCs and tdEVs are promising surrogate markers in diagnosing PCa [21,22,23]. In this study, the authors enumerate both the CTCs and tdEVs in the mCRPC samples. The authors show that ≥5 CTC/7.5 mL was detected in the peripheral blood (PB) in 42 out of 57 samples eligible for CTCs counting using the CellSearch^®^ system (Menarini Silicon Biosystems, Bologna, Italy) and correlated with a lower OS rate. Similarly, ≥20 tdEVs /7.5 mL of PB was detected in 38 out of 46 samples eligible for tdEVs counting and associated with a lower OS rate. Both CTCs and tdEVs were detected in 29/46 samples, suggesting a strong positive correlation between the CTCs count and the tdEVs count, which was confirmed by the Spearman’s rank correlation coefficient (rs) of 0.841, with statistical significance of *p* < 0.001.

## 2. Conclusions

In this study, the authors examined the gene expression and DNA methylation variations in several markers between the CTCs and plasma-derived exosomes in mCRPC and HDs and directly compared those markers. They show that the EpCAM-positive CTCs, CK-19, PSMA, TWIST1 expression, and GSTP1 methylation were significantly associated with a lower OS rate and an increased the risk of death; though, in exosomes, the CK-8 expression and GSTP1 and RASSF1A methylation statuses were significantly related with a lower OS rate and an increased risk of death. The study also highlights the potential diagnostic value of CTCs and tdEVs enumeration as surrogate markers in PCa.
